# The Vaccination Question

**Published:** 1895-03-16

**Authors:** 


### MEDICAL PROGRESS AND HOSPITAL CLINICS.

[*The Editor will be glad to receive offers of co-operation and contributions from members of the profession. All letters should be addressed to* THE EDITOR, THE LODGE, PORCHESTER SQUARE, LONDON, W.]

### THE VACCINATION QUESTION.

EXCEPTING for a certain unaccustomed moderation of language, this little book[1] does not differ very greatly from most of the anti-vaccination literature of the day. While the style is more subdued, the usual accusations of bias, and interested motives, and so forth are urged against those who differ from Mr. Hutton as to the value of vaccination. It is a practice, we are told, which is firmly established "by interest," as well as by law and custom, and as to small-pox, "it would almost seem that the special prominence given to it now is due to the interested advocacy of prophylactics against it;" while, regarding the Medical Department of the Local Government Board, Mr. Hutton writes as follows: "As I happen to know, a mouthpiece of the medical officials of the Local Government Board may ultimately feel ashamed of *the lies which he has had to tell at their dictation.*" The italics are ours. It must be rather startling to Mr. Asquith to be told that some member of Parliament, who had been thought worthy by the Premier of the day to hold high office in his ministry—probably either the Presidentship or the Parliamentary Secretaryship of the Local Government Board—should have been a creature so utterly beneath contempt as to tell lies to Parliament, at the "dictation" of official servants and subordinates.

Mr. Hutton is good enough to allow, however, that doctors are not all alike untruthful and untrustworthy on the subject of vaccination. It appears, indeed, that in certain instances a medical man has approached the question "with his mind as free as possible from bias." It is, of course, sheer accident—bias on Mr. Hutton's part can have nothing to do with it—that these men, saved as by fire from condemnation, belong to the exceedingly minute minority who agree with Mr. Hutton in opposing vaccination.

Mr. Hutton informs Mr. Asquith that there is nothing to prevent any educated layman from learning all that is scientifically known about physiology, pathology, and pharmacy, and that in any one special field of inquiry a layman may easily be better informed than a professional man, who has a wider range of study to pursue. This being so, why did not Mr. Hutton take the trouble, before going into print, to learn all that a layman can so easily learn about the subject? He tells us, for example, that doctors operate by *subcutaneous* insertion of the cow-pox virus, as if this were not exactly what every medical man carefully avoids. With singular intolerance he intimates that, so far as he knows, "no one" who has studied the history and pathology of vaccination "has retained his faith in its alleged power." He bases a statement that in the last century small-pox prevalence was not "universal," on the fact that in a town in New England the interval between two particular epidemics was 19 years. He says that small-pox inoculation "became the rage" in England immediately after its introduction by Lady Mary Wortley Montagu and its adoption by the Princess of Wales; whereas he could have learned from the "unbiassed" teaching of Professor Crookshank that less than 900 inoculations were recorded in the whole country in the first eight years, and that then the practice almost disappeared for a long period. With the easy assurance which is born of half knowledge he tells us it is "undoubtedly true" that inoculation "destroyed more lives than it saved," though there is no question in the history of small-pox more doubtful. He says, "we talk of small-pox inoculation as if it were a uniform practice." Who are the "we" for whom he speaks? He says "it may be admitted" that in special cases and places small-pox inoculation "might still be resorted to with advantage." Who "admits" this excepting Professor Crookshank, with whom the proposal originates? Following Dr. Creighton, he repeats to Mr. Asquith the statement that Jenner was not honest, and without any reference to the *Lancet's* article on the "cuckoo" story (readily available to a layman, biassed or otherwise) he accepts without demur the previous reckless assumptions that had in this connection been made against Jenner. He goes on to show the value of a layman's study of the subject by assuming that the term "swine-pox" belonged to a disease of pigs. In defiance of easily ascertainable historical fact, he tells Mr. Asquith that all the medical men at a local meeting with Jenner denied, from their own experience, the protective power of cow-pox. On the next page, having stated his opinion, or Dr. Creighton's, that the term cow-pox "had reference not to small-pox but to great-pox," he commits himself to the astounding mis-statement that this alleged analogy "was popularly and correctly discerned" in the early days of vaccination, and he adds that it "is now certainly established beyond all reasonable doubt" that "cow-pox bears no pathological relation to small-pox." On one and the same page he makes the two statements (1) that "even the warmest advocates of the practice admit that the protection afforded by vaccination extends only for a period variously reckoned at from one to ten years," and (2) that "in every case of the failure of vaccination to secure immunity we have a distinct contradiction of the theory of its prophylactic virtue." Thus the occurrence of small-pox after an interval of say sixty or seventy or eighty years from vaccination is, according to Mr. Hutton, a contradiction of a theory which attributes to vaccination only from one to ten years' prophylactic power. It would be hard to say which of Mr. Hutton's two statements is the further from the truth, but it is psychologically interesting that he should give expression to both of them, and that almost with the same stroke of the pen. Of course, the medical theory is that neither vaccination nor any other recognised pro-

[1] "The Vaccination Question." A letter addressed by permission to the Right Hon. H. H. Asquith, Q.C., M.P., by A. W. Hutton, M.A., Librarian to the National Liberal Club. (Methuen and Co., 36, Essex Street, W.C. Price 1s. 6d.)

tection against small-pox, though extending far beyond ten years, is to be relied on as either absolute or permanent, and that the occurrence of small-pox after vaccination is no contradiction of the doctrine of prophylaxis, any more than is the occurrence of scarlet fever after scarlet fever, or small-pox after small-pox.

Mr. Hutton's book runs to some one hundred and thirty pages. The above notes refer to the first twenty pages only. He begins by arguing that a layman is thoroughly competent to discuss and decide the vaccination question, and in the course of the first few pages he demonstrates the inability of one particular layman to do anything of the kind. He seems to have read most of what Dr. Creighton and Mr. Crookshank have written, but to be quite wanting in the power of separating the wheat from the chaff, if indeed, it has ever struck him to inquire whether the writings of these two authors contain anything else than pure grain.

Leaving history and pathology, and coming to statistics, the author has very great doubt as to the value of much statistical material, but once more the doubt appears to apply to statistics adduced in support of vaccination. He lauds (at the same time that he misquotes the title of) Mr. A. Russell Wallace's anti-vaccination pamphlet on registration statistics, but he scouts the honesty and the validity of much that is written on the other side. Indeed, even Pasteur—his treatment of rabies bearing some analogy to vaccination—falls at once under suspicion, as indicated in this way: "Of course these are the statistics issued by his friends and employés . . ." In these circumstances it is fortunate that Mr. Hutton provides us with two pages or tables of figures which may be taken as an example of what he considers proper statistics, prepared by a perfectly unbiased person—to wit, himself. The lesson which is to be deduced from the two tables is to be got by comparing them. Table 1 is described as "showing the 22 towns or districts (excluding London) which have suffered most from small-pox during the last 7½ years, and showing also how far they neglected vaccination, 1885-90." We would expect that Table 2, for comparison with this, would show the 22 towns, &c., which have suffered least from small-pox in the same period. Instead, Table 2 shows the 22 towns in which vaccination was most neglected in 1885-90, and how far they have suffered from small-pox during the last 7½ years. Letting that pass, however, an essential question is: Assuming that the current opinions of the medical profession regarding vaccination are correct, what exactly can be the influence of neglect of infantile vaccination in 1885-90 on deaths of adults from small-pox in 1887-94? We have already noted that Mr. Hutton informs Mr. Asquith that what medical men claim for vaccination is a protection lasting from 1 to 10 years. This is a singularly inadequate statement, but it is correct in so far as it indicates that medical men are of opinion that the protective power of vaccination diminishes with advancing age. Now the neglected vaccination which appears in Mr. Hutton's table is neglected vaccination under three months of age, beginning in 1885. Obviously, therefore, such neglect could hardly be responsible for deaths from small-pox of persons who had been born previous to 1885. The deaths, indeed, in order to have direct relation to the vaccination default, should only be deaths in *young children;* and yet Mr. Hutton includes in his table the deaths which occurred *in every period of life.* Again, any prevalence of small-pox in 1887-94 appears to have been confined to the groups of years at the beginning and end of the period, there being almost none in the three years 1889-91. Mr. Hutton gives no information as to how far the towns were exposed to infection in the earlier period of prevalence, but it is open to suspect that had the towns in Table 2 been more seriously invaded, the vaccinations would have rapidly increased, as they are apt to do under small-pox prevalence combined with practically optional vaccination. Regarding the last two years of the period, it is really surprising to find that Mr. Hutton should present his readers with a table in which the deaths from small-pox were "not yet to hand" for no less than eleven of the twenty-two ill-vaccinated towns, while for comparison, the twenty-two towns which suffered most from small-pox have the figures supplied for all but four or five of them. No doubt Mr. Hutton could not get the omitted data; but why, then, as a critic of statistics, did he ever allow himself to publish so ridiculously defective a tabulation?

The character of the whole argument involved in these two tables may, perhaps, be illustrated by a parallel argument regarding the use or uselessness of blankets as preventives of death from cold or exposure. Exposure to vicissitudes of temperature is apt to kill a greater or less number of persons subjected thereto. All people are agreed as to this. Also, there is general agreement as to infants being more liable to die of cold than other persons, and there is a belief shared in by the majority of people that blankets (even when worn and old) tend to secure individuals provided with them against dangerous vicissitudes of temperature. Let us suppose, however, that a minority have observed that children are occasionally smothered by blankets, and that they further proceed to contend that blankets (new as well old) are useless as security against vicissitudes of temperature. Notwithstanding this, persons who, either through acceptance of these opinions, or through indifference and neglect, fail to provide their children with blankets against the severities of frost and exposure, are punished by law for omissional cruelty to their offspring. The minority, accordingly, forms an Anti-Blanket League, and Mr. Hutton becomes its spokesman, and produces two tables of statistics, as to which he argues as follows:—

*Table* 1.—In several separate localities, during seven or eight months of a given period, there were found dead in bed from exposure to cold differing proportions of the populations (adolescents and adults mainly), most of which persons are believed to have been provided at some time of their lives (gratuitously or otherwise) with blankets. "But, obviously," says Mr. Hutton, "the differing amounts of deaths from exposure to cold in these several localities can have had no relation whatever to the presence or absence of blankets among persons succumbing in this way, seeing that the number of newly-born infants in these several localities provided (gratuitously or otherwise) with blankets during six months of practically the same period, differed widely, and bore no relation whatever to the number of adolescents and adults dying of exposure to cold."

"*Table* 2.—Again, in every one of several other and separate localities unusually few newly-born infants were, during the same six months of the period in question, provided with blankets; nevertheless, in hardly any of these other localities was there, in seven to eight months of practically the same period, any considerable total of deaths (made up in unstated numbers of infants and adolescents and adults) referred to exposure to cold. Obviously, therefore," says Mr. Hutton, "blankets may safely be dispensed with, for the circumstance that cold did not prevail to any appreciable extent in these localities during the particular period is too trivial to be taken account of in this connection."

(*To be continued.*)

